# Implicit gender-career bias in postgraduate medical training still exists, mainly in residents and in females

**DOI:** 10.1186/s12909-021-02694-9

**Published:** 2021-05-02

**Authors:** Maud Kramer, Ide C. Heyligers, Karen D. Könings

**Affiliations:** 1 School of Health Professions Education (SHE), Faculty of Health, Medicine and Life Sciences, Maastricht University, Maastricht, The Netherlands; 2 Department of Orthopaedic Surgery and Traumatology, Zuyderland Medical Centre, Heerlen, The Netherlands

**Keywords:** Implicit bias, Gender-career bias, Stereotype, Gender, Career ambition, Resident, Clinical trainer, Postgraduate medical training

## Abstract

**Background:**

More and more female residents enter postgraduate medical training (PGMT). Meanwhile, women are still underrepresented in academic medicine, in leadership positions and in most surgical specialties. This suggests that female residents’ career development may still be negatively impacted by subtle, often unconscious stereotype associations regarding gender and career-ambition, called implicit gender-career bias. This study explored the existence and strength of implicit gender-career bias in doctors who currently work in PGMT, i.e. in attending physicians who act as clinical trainers and in their residents.

**Methods:**

We tested implicit gender-career bias in doctors working in PGMT by means of an online questionnaire and an online Implicit Association Test (IAT). We used standard IAT analysis to calculate participants’ IAT D scores, which indicate the direction and strength of bias. Linear regression analyses were used to test whether the strength of bias was related to gender, position (resident or clinical trainer) or specialty (non-surgical or surgical specialty).

**Results:**

The mean IAT D score among 403 participants significantly differed from zero (D-score = 0.36 (SD = 0.39), indicating bias associating male with career and female with family. Stronger gender-career bias was found in women (β_female_ =0 .11; CI 0.02; 0.19; *p* = 0.01) and in residents (β_resident_ 0.12; CI 0.01; 0.23; *p* = 0.03).

**Conclusions:**

This study may provide a solid basis for explicitly addressing implicit gender-career bias in PGMT. The general understanding in the medical field is that gender bias is strongest among male doctors’ in male-dominated surgical specialties. Contrary to this view, this study demonstrated that the strongest bias is held by females themselves and by residents, independently of their specialty. Apparently, the influx of female doctors in the medical field has not yet reduced implicit gender-career bias in the next generation of doctors, i.e. in today’s residents, and in females.

## Background

Although the percentage of female doctors is increasing worldwide [1], women are still underrepresented in academic medicine, in leadership positions [2, 3] and in historically masculine surgical specialties [4–6]. This suggests that female doctors’ career chances and choices may still be negatively impacted by subtle, often unconscious stereotype associations regarding gender and career ambition, also called *implicit gender-career bias* [7–11]. Implicit gender-career bias held by attending physicians who act as clinical trainers in postgraduate medical training (PGMT) has been reported to negatively influence the supervision, mentoring and assessment of their female residents [12–14]. In times when female residents dominate the trainee groups in most specialties, it seems highly important to create insight into the current existence and strength of gender-career bias in PGMT.

Implicit gender-career bias is unintentionally learned due to historically and socioculturally embedded stereotypical gender role expectations: women are communal (e.g., warm and concerned about the wellbeing of others) and should therefore give priority to care for the family, whereas men are agentic (e.g. competitive and ambitious) and should primary perform at work [15–17]. People are not aware of implicit bias, which might actually be in contrast to their explicit thoughts [18, 19] and might unconsciously affect their understanding and their behavior (in actions and decisions) [15]. As a consequence, implicit gender-career bias can be persistent, even if the work environment is increasingly dominated by women [20].

Gender bias can result in the so-called stereotype threat for female residents, which means that they are afraid of confirming the negative stereotype perception of “women as a group” [9–20]. Stereotype threat can negatively affect female residents’ well-being [21], self-efficacy [22] and functioning [23, 24]. Mainly in surgical specialties that are known for their gender imbalance and masculine work culture, female doctors have been reported to experience difficulties resulting from gender bias due to subtle or not so subtle discrimination, which can lead to feelings of not fitting into their work environment [25–27]. As a consequence, many women tried to adapt their identity to fit the surgical work-environment [26, 27]. At the same time, high attrition rates are found among female surgical residents [28]. These earlier findings may suggest strong implicit gender-career bias in both male and female doctors in surgical specialties.

The existence of implicit gender-career bias has been reported before among health care professionals in general [29]. According to Salles et al. (2019), female health care professionals in general held stronger gender-career bias than males [29]. Slightly different types of implicit gender bias were also confirmed in different groups of doctors: gender-specialty bias was found in surgeons, and gender-leadership bias was found in residents and in academic faculty members [29–31]. However, these studies found that male residents and male academic faculty members in particular held stronger gender-leadership bias than their female colleagues [30, 31]. In the male-dominated specialty of emergency medicine, the relation between male gender and gender-leadership bias was even stronger [31]. In contrast, Salles et al. (2019) found no gender differences in gender-specialty bias in general surgery, which also is a male-dominated specialty [29]. Thus, the association between gender and implicit gender bias seems not to be clear yet and may differ between populations (e.g. between doctors within a specialty) and between slightly different types of gender bias.

Gender-career bias in residents and their clinical trainers in PGMT is yet unknown but is likely to impact the quality of clinical training. While the above mentioned studies all used a validated test for directly measuring implicit bias, the Implicit Association Test (IAT) [32, 33], this tool has never been used to investigate gender-career bias in these stakeholders in PGMT. According to previous studies, clinical trainers whose behavior is influenced by gender bias can transfer this bias to the younger generation of residents in PGMT, thus preserving bias in the medical field [24, 34]. Creating awareness of their possible bias can help clinical trainers and residents to reduce the effects of this bias on clinical supervision at the workplace and on residents’ career chances and choices.

The aim of this study was to investigate the direction and strength of implicit gender-career bias among doctors working in PGMT, and to explore whether this bias is related to a doctor’s position (*clinical trainer* or *resident*), gender or specialty (*a surgical specialty* versus *a non-surgical specialty*).

## Methods

### Participants

The Netherlands is divided in eight ‘resident training regions’ based on geographic location of the eight medical universities. Two comparable resident training regions, regions Southeast and Northeast, were selected to recruit participants for the present study. These regions are both large in size, both borderline and relatively rural regions, and both contain one academic centre surrounded by multiple general teaching hospitals and some other affiliated medical teaching centres (f.e. rehabilitation centres and psychiatric clinics).

The health care institutions in these regions were separately approached for participation in our study. Seven health care institutions (one academic centre, two large general teaching hospitals, three smaller general teaching hospitals and one rehabilitation centre) in region Southeast and three health care institutions (one academic centre and two large general teaching hospitals) in region Northeast joined our study.

Physicians and residents from all specialties working in PGMT settings in the participating health care institutions were invited to participate in an online survey and a linked online IAT. We included participants from all specialties, but excluded respondents from clinical chemistry, clinical physics and pharmacy, as they are not physicians, and their educational background and daily work activities differ too much from residents and physicians in the other specialties. Furthermore, we included both ‘residents-in-training’ and ‘residents-not-in-training’ as ‘residents’ in this study. Before PGMT, most doctors in the Netherlands already do clinical work as ‘residents-not-in-training’ to increase their likelihood of becoming accepted for the resident training program of their preference. ‘Residents-in-training’ and ‘residents-not-in-training’ are peers who have similar daily clinical work activities and are supervised by the same medical specialists (or clinical trainers) in the same work environment.

### Procedure

Physicians and residents were invited by e-mail to voluntarily and anonymously complete the online questionnaire and the online IAT. The e-mails were sent to potential participants by each participating health care institution after approval by the management. No response rate was calculated since the mailing procedure differed between institutions (e.g. central distribution using a mailing list for all specialties at once, or via the program coordinators of each specialty separately), which made it impossible to ascertain the total number of invited physicians and residents.

Two reminders were sent within a period of 2 months. After 2 months, data collection stopped by closing the online questionnaire and IAT. In the Southeast resident training region, data were collected from March 2017 to May 2017. In the Northeast resident training region, data were collected from May 2019 to July 2019. Ethical approval of this study was obtained from the NVMO (the Dutch Society of Medical Education) Ethical Review Board, document number 743.

### Measures

#### Survey of individual characteristics and work environment

The survey comprised five questions about participants’ gender (“what is your gender?”), age (“what is your age?”), type of specialty (“In which medical specialty do you work?)”, position (“what is your position (resident-not-in-training/ resident-in-training/ specialist)?”, work location (“In which health care institution do you work?”).

#### Implicit association test

The IAT quantifies the direction and strength of implicit bias and is characterized by its high internal consistency and predictive validity [32, 33, 35, 36]. The gender-career IAT used in this study assesses the implicit association in people’s minds between two pairs of concepts [19]: male-and-career and female-and-family (stereotype pairings) versus male-and-family and female-and-career (non-stereotype pairings). Participants were instructed to sort words that appeared on the computer screen, also called *stimuli*, as quickly as possible into different categories. Stimuli were male and female nouns, career-related words and family-related words (Table 1).

**Table 1:** Stimuli per category used in the IAT

Stimuli in category “male”	Stimuli in category “female”	Stimuli in category “career”	Stimuli in category “family”
Male	Female	Ambitious	Home
Mister	Missus/ miss	Promotion	Leisure
Sir	Madam	Working overtime	Friends
Young man	Young lady	Being in conference	keeping house
He	She	Salary	Family
Him	Her	Medical specialist	Children
		Trainer/ supervisor	Cooking

Note. Stimuli (= words per category) are presented here in the English translation of the Dutch words used in the study

The IAT is based on the logical premise that easier pairings (faster response and fewer errors) are more strongly implicitly associated in the participant’s mind than more difficult parings (slower response and more errors) [19, 32].

For the online IAT, we used Inquisit 5 Web Edition. The version used in this study was an adapted Dutch version of the online gender-career IAT hosted by Harvard [37]. The IAT was built up in accordance with the guidelines of Nosek et al. (*2007*) and contained seven blocks [33]. The blocks differed in their instructions for using two marked response keys, the ‘e’ key on the left and the ‘I’ key on the right of the keyboard, to classify stimuli that appear in the middle of the computer screen into two different categories (male or female/ career or family; in blocks 1, 2 and 5) or paired categories (male-and-career/ female-and-family/ male-and-family/ female-and-career; in blocks 3, 4, 6 and 7) on the left and right side of the screen. In blocks 3 and 4, the paired category “male-and-career” shared one response key and the paired category “female-and-family” shared the other response key, in order to assess participants’ response times regarding stereotypical pairings. In blocks 6 and 7, participants’ response times regarding the non-stereotypical pairings were assessed: “female-and-career” shared the same response key, as did “male-and-family”. The blocks 3, 4 and 6, 7 were counterbalanced: half of the participants first sorted the stereotypical pairings, and the other half first sorted the non-stereotypical pairings. Participants were instructed to remain concentrated and undisturbed while completing the IAT and to do so as quickly as possible. If words were categorized incorrectly, an error indication appeared (red “X”) and the participant had to fix the error by pressing the correct response key before continuing to the next words. Results of the online IAT per participant were automatically registered and stored in an online database.

For each participant, the scoring algorithm as described by Greenwald et al. (2003) was used to calculate the IAT D score [35]. The differences in response times (response latencies) of the non-stereotype pairings and the stereotype pairings of the test blocks 4 and 7 was divided by the standard deviation (SD) of all response latencies (from blocks 4, 5, 6, and 7). The IAT D score is an effect size measure ranging from − 2 to + 2. Higher, positive values indicate a stronger association (bias) of the stereotype parings. For example: a D-score of 0.5 means that a participant was 0.5 s faster in sorting stereotype pairing than in sorting the non-stereotype pairings. Lower, negative values indicate a stronger association of the non-stereotype pairings.

### Statistical analysis

We examined the overall IAT D-score of the participants and tested differences in IAT D-scores among participants considering their gender, position (resident or physician) and specialty (surgical or non-surgical specialty) in a multiple linear regression model, and corrected for each participant’s age and the region of sampling. Correcting for age is important to determine the impact of position only, because physicians are mostly older than residents, and earlier studies showed significantly stronger implicit gender bias with increasing age [29, 30, 33], hence the relation between age and position might interfere with the strength of gender bias. Lastly, we added the interaction terms gender*specialty and gender*position to the model to explore if gender differences varied per specialty and position, respectively. For the regression analyses, only data of participants without missing variables were included. We used *p* < .05 as statistical significance level in all analyses. Analyses were performed in IBM SPSS Statistics 26.

## Results

A total of 432 doctors completed the online questionnaire and the IAT. We excluded 19 participants from the analyses because of working in one of the technical specialties (clinical chemistry, clinical physics or pharmacy). Furthermore, 10 participants were excluded because not filling in their specialty. Among the remaining 403 participants, 223 were from region Southeast (55%), 242 were women (60%) and 184 were residents (46%; 148 of them were resident-in-training and 36 were residents-not-in-training) and 133 worked in surgical specialties (33%). The mean age of the participants was 38.78 (SD = 10.82); the mean age of the residents was 30.22 (SD = 3.20) and of the physicians 45.96 (SD = 9.68).

The mean IAT D score was 0.36 (SD = 0.39), which means that all the participants associated the stereotypical pairings “male-and-career” and “female-and-family” more strongly than the non-stereotypical pairings “male-and-family” and “female-and-career”. Mean IAT D score in female doctors was 0.39 (SD = 0.39) compared to 0.31 (SD = 0.38) in male doctors (t (401) = 2.07; *p* = 0.04). Mean IAT D score in residents was 0.40 (SD = 0.36) while being 0.33 (0.41) in clinical trainers (t (401) = 1.47; *p* = 0.14). Mean IAT D score was 0.36, both in surgical doctors (SD = 0.37) and in non-surgical doctors (SD = 0.39) (t (401) = −0.11; *p* = 0.91).

Table 2 shows descriptives of IAT-D scores by position and specialty for male and female participants separately. Independent *t*-tests comparing male and female in different positions and specialties showed that only gender differences in non-surgical specialties were significant (t (268) = 2.40; *p* = 0.02). All other comparisons were non-significant.

**Table 2:** Descriptives of IAT D-scores by position and specialty for male and female doctors separately

	IAT D-score	
	Male M (SD)	Female M (SD)
**Position:** Residents	0.36 (0.42)	0.41 (0.34)
Clinical trainers	0.29 (0.39)	0.32 (0.37)
**Specialty:** Surgical doctors	0.35 (0.34)^a^	0.37 (0.40)^a^
Non-surgical doctors	0.28 (0.40)	0.40 (0.38)

Note ^a^T-test analyses showed significant differences between non-surgical female doctors’ and non-surgical male doctors’ mean IAT D scores

The results of the multiple regression analysis (*N* = 403) are shown in Table 3.

**Table 3:** Results of multiple regression analyses testing the relation between IAT D-scores and gender, position specialty, corrected for region and age

	Multiple regression analysis	
	B (95% CI)	***p***-value
**Gender:**		0.01
Female	0.11 (0.02; 0.19)	
Male	*reference*	
**Position:**		0.03
Resident	0.12 (0.01; 0.23)	
Clinical trainer	*reference*	
**Specialty:**		0.96
Surgical^a^	0.00 (−0.08; 0.08)	
Non-surgical^b^	*reference*	
**Region:**		0.03
Southeast	0.08 (0.01; 0.16)	
Northeast	*reference*	
**Age**	0.01 (0.00; 0.01)	0.04

Note

^a^Surgical specialties included general surgery, cardiothoracic surgery, plastic surgery, orthopedic surgery, neurosurgery, urology, ear-nose-throat, maxillofacial surgery, ophthalmology and obstetrics & gynecology

^b^Non-surgical specialties included cardiology, internal medicine, respiratory medicine, emergency medicine, pediatrics, intensive care medicine, neurology, dermatology, rheumatology, psychiatry, rehabilitation medicine, sport medicine, anesthesiology, pathology, radiology, radiotherapy, medical microbiology and clinical geriatrics

Significant p-value (*p* < 0.05)

Model *R* square = 0.034

Both gender (*p* = 0.01) and position (*p* = 0.03) were significantly related to IAT D scores: female participants’ IAT D score were 0.11 points higher compared to male participants, and residents’ IAT D score were 0.12 points higher compared to clinical trainers. IAT D scores did not significantly differ between surgical and non-surgical specialties (*p* = 0.96). The interactions between gender*specialty and between gender*position were non-significant (*B* = −0.10 [−0.27;0.06], *p* = 0.21 and *B =* −0.00 [−0.16; 0.16,] *p* = 0.97, respectively). Thus, the relation between gender and IAT D score did not significantly vary by specialty or by position. The full regression model is included in the Appendix.

## Discussion

To our knowledge, this is the first study that used the IAT to measure the direction and strength of “implicit gender-career bias” in residents and clinical trainers in PGMT settings. We found that all doctors involved in PGMT held implicit bias regarding gender and career-ambition in the stereotypical direction: they associated male more strongly with career and female more strongly with family than female with career and male with family. Independently of their specialty, females held stronger gender career-bias than their male colleagues, and residents held stronger gender career-bias than clinical trainers.

The overall strength of gender-career bias in the stereotypical direction found in our study is comparable to earlier findings in the previous study of Salles et al. (2019) on a broader group of “diagnosing and treating health care professionals” [29]. Although female residents in their numbers already dominate the current “trainee group” in PGMT within some specialties [5], residents themselves, irrespective of their specialty, held even stronger bias than their clinical trainers who entered the field when this was still dominated by male doctors. As we corrected for participants’ age, we may assume that stronger bias in residents is purely an effect of their position, i.e. being a resident. This finding underlines that implicit bias is persistent and lags behind societal changes [15, 20, 38]. Despite the influx of female doctors in the medical field, major cultural transformation seems not to have occurred yet [39, 40]. Implicit gender-career bias is still present within the next generation of doctors, i.e. the current residents in PGMT.

It might sound irrational that female doctors hold stronger implicit gender-career bias than their male colleagues. One might expect that women who aspire to a career as a doctor would be intrinsically convinced of women’s career ambitions. Indeed, female surgeons reported low explicit (conscious) gender bias when they were asked directly [29]. However, we measured *implicit* bias instead of *explicit* bias. Women’s implicit bias might be in contrast to their explicit thoughts, unconsciously resulting from deep-rooted stereotypical perceptions [15, 16] or triggered by negative experiences of subtle gender bias or discrimination in their work environment [27]. Male doctors are less aware of gender issues in the medical field [24, 41–43]. A recent study showed that male academics, regardless of their career stage, did not show evidence of gender stereotyping in judging career commitment of their colleagues [44]. This might explain our findings that male doctors’ implicit gender-career bias was less strong than their female colleagues’ bias, independently of their position.

Strong implicit gender-career bias in female doctors might be a critical contributor to gendered career paths in medicine. Literature outside the medical field described the phenomenon that female employees seem to underestimate or ‘devalue’ their own professional ambitions, agency, and career commitment, whereas young men do not [45]. In the medical field, male doctors easily find “sponsors” and are encouraged to run for elected leadership positions [8, 46]. Female doctors mentioned “lack of support and mentors” as barriers to strive for these positions [10, 23, 46]. As a result, female residents searching for female role models recognize gender disparities in these top positions, which again might intensify implicit gender-career bias [43]. Thus, gendered career paths and persistent implicit gender bias might be a vicious circle, mainly recognized by female doctors [6]. Moreover, lack of gender diversity in faculty may further explain stronger implicit gender-career bias in female doctors – independently of their position – in our study.

Surgical specialties are mainly male-dominated and are known for their masculine work culture characterized by strong stereotypes [47]. Moreover, women experience both a lack of same-gendered role models and difficulties to position themselves in or adapt themselves to this work environment [26, 34]. Especially in surgical specialties, including the only female-dominated specialty gynaecology and obstetrics, female residents and physicians experience high levels of gender bias in the work environment [27]. Therefore, we expected higher implicit gender-career bias in all doctors working in surgical specialties, and particularly in female surgical doctors. However, our results did not confirm this expectation, as we found no differences in gender-career bias between surgical and non-surgical specialties, nor were gender differences in strength of bias impacted by specialty.

Our findings that implicit gender-career bias does not seem to be stronger in surgical doctors than in non-surgical doctors are in line with earlier studies that identified gender stereotype perceptions, such as “men are better physicians”, across residents in all specialties [21]. Although female doctors’ negative experiences of explicit gender bias in surgical specialties has been extensively described in previous studies [21, 26, 27, 34], their own implicit gender-career bias does not seem to be stronger compared to female doctors in non-surgical specialties. It could be hypothesized that also the non-surgical work culture still is highly masculine, despite the work population is already more female-dominated. This might trigger implicit gender-career bias in female non-surgical doctors in the same degree as in their female surgical colleagues and might explain why gender differences in the strength of gender-career bias were not impacted by specialty in our study.

Our findings have practical implications for PGMT. As all the doctors in our study held implicit gender-career bias, gender bias needs attention in PGMT. Doctors working in PGMT settings should be aware of the possible presence of implicit gender bias and recognize that this bias may unintentionally influence their own behaviour and actions [48]. Awareness and recognition of gender bias at the individual level is the first critical step in reducing the effects of gender-career bias [15, 20, 49].

Although clinical trainers hold lower bias than their residents, subtle bias in physicians can already reduce the effectiveness of the clinical training, particularly for the large group of female residents. Physicians must recognize that implicit gender bias might influence their perception and evaluation of residents [12–14]. They should be aware of gender differences in residents’ learning styles and in residents’ self-evaluations, as female residents have a stronger tendency to downgrade their own functioning than male residents [50, 51]. Female clinical trainers must be stimulated to function as mentors for their same-gender residents [49, 51]. Furthermore, clinical trainers of both genders should encourage female residents who aspire to a career in prominent educational or leadership positions [49].

Single educational interventions can create awareness of bias and have been proven to positively limit individuals’ bias in the short term [8, 30]. However, they will not reduce the effects of bias in PGMT in the long term. Therefore, “bias awareness” should be integrated as an important recurring topic in educational activities of both residents and their clinical trainers in order to address this bias [48].

Next, in order to break the vicious circle of gendered career paths and persistent implicit gender bias [6], striving for gender diversity throughout the whole organization should be a priority on the agenda of medical institutions [52, 53] . Future female leaders in the medical field can function as critical actors and initiate long-term policies for cultural transformation to a more ‘inclusive work culture’ and thereby decrease implicit gender-career bias in the medical profession [39, 54, 55].

Our study has several limitations. It was limited by the relatively small sample size of 432 participants and the unknown response rate of doctors per health care institution. We also do not know which individuals decided to participate in the study and if they are adequately representing the population of residents and physicians. Furthermore, the use of the IAT as a metric is limited, but it is the current golden standard for measuring implicit bias. We used the scoring algorithm as described by Greenwald ea. (2003) to calculate the IAT D score as our measure for implicit bias [35]. More recent literature reported that this IAT D score possibly underestimates the real degree of implicit bias [56]. This assumption might indicate that the implicit gender-career bias in doctors is even stronger than the bias we found in our study. Furthermore, in the analyses, we corrected for differences in bias among the two residency training regions of data collection. We did not expect an influence of these regions on doctors’ implicit gender bias, as the two regions are quite similar in the size and distribution of health care institutions (one university hospital and multiple teaching hospitals per region). Contrary to our expectation, we found differences in bias among the two regions, which we cannot explain, but corrected for. Speculating, culture differences at organizational level in three relatively large health care institutions in region Northeast compared to seven (both larger and smaller) institutions in region Southeast might have caused the differences in bias among the two regions. Differences in bias between regions and institutions in relation to culture could be the focus of future research. On country level, differences in gender norms and in gender (in) equality might be related to individuals’ implicit gender bias. The Netherlands is an egalitarian country with already a large female workforce in the medical field [1, 57, 58]. Therefore, it could be hypothesized that in other contexts, doctors’ implicit gender-career bias might be even stronger and that gender differences in implicit gender-career bias might be larger in respectively less egalitarian countries and in countries with less share of female doctors.

Our findings underscore the need for increased attention to doctors’ implicit gender bias, which might be in contrast to their explicit thoughts and experiences. Therefore, we recommend future studies to focus on studying the effects of educational interventions and on formulating guidelines for reducing the influence of gender-career bias in PGMT. Furthermore, effects of bias-reducing interventions need to be evaluated at the implicit level, in addition to the explicit level of conscious perceptions and beliefs. Future longitudinal studies could also follow up on the long-term developments of implicit gender-career bias after curricular changes and institutional policy changes.

## Conclusions

This study may provide a solid basis for explicitly addressing implicit gender-career bias in PGMT. The general understanding in the medical field is that gender bias is strongest among male doctors in male-dominated surgical specialties. Contrary to this view, this study demonstrated that the strongest bias is held by females and residents, independently of their specialty. Apparently, the influx of female doctors in the medical field has not yet reduced implicit gender-career bias in the next generation of doctors, i.e. today’s residents, and in females. Awareness and recognition of the existence of implicit gender bias is the first critical step in reducing the effects of gender-career bias in the medical field.

## Data Availability

The datasets used and/or analysed during the current study are available from the corresponding author on reasonable request.

## Appendix

**Table 4:** Results of multiple regression analyses testing the relation between IAT D-scores and gender, position specialty, corrected for region and age: full model including non-significant interaction terms

	Multiple regression analysis	
	B (95% CI)	***p***-value
**Gender:**		0.04
Female	0.14 (0.01; 0.28)	
Male	*reference*	
**Position:**		0.04
Resident	0.13 (0.01; 0.25)	
Clinical trainer	*reference*	
**Specialty:**		0.43
Surgical^a^	0.04 (0.06; 0.15)	
Non-surgical^b^	*reference*	
**Region:**		0.03
Southeast	0.08 (0.01; 0.16)	
Northeast	*reference*	
**Age**	0.01 (0.00; 0.01)	0.05
**Gender*position**	−0.00 (−0.16; 0.16)	0.97
**Gender*specialty**	−0.10 (−0.27; 0.06)	0.21

Note

^a^Surgical specialties included general surgery, cardiothoracic surgery, plastic surgery, orthopedic surgery, neurosurgery, urology, ear-nose-throat, maxillofacial surgery, ophthalmology and obstetrics & gynecology

^b^Non-surgical specialties included cardiology, internal medicine, respiratory medicine, emergency medicine, pediatrics, intensive care medicine, neurology, dermatology, rheumatology, psychiatry, rehabilitation medicine, sport medicine, anesthesiology, pathology, radiology, radiotherapy, medical microbiology and clinical geriatrics

Significant p-value (*p* < 0.05)

Model *R* square = 0.038

## Abbreviations

PGMT: Postgraduate medical training
IAT: Implicit Association Test
NVMO: Dutch Society of Medical Education
